# First effects of rising amyloid-β in transgenic mouse brain: synaptic transmission and gene expression

**DOI:** 10.1093/brain/awv127

**Published:** 2015-05-16

**Authors:** Damian M. Cummings, Wenfei Liu, Erik Portelius, Sevinç Bayram, Marina Yasvoina, Sui-Hin Ho, Hélène Smits, Shabinah S. Ali, Rivka Steinberg, Chrysia-Maria Pegasiou, Owain T. James, Mar Matarin, Jill C. Richardson, Henrik Zetterberg, Kaj Blennow, John A. Hardy, Dervis A. Salih, Frances A. Edwards

**Affiliations:** 1 Department of Neuroscience, Physiology and Pharmacology, UCL, Gower St, London WC1E 6BT, UK; 2 Institute of Neuroscience and Physiology, Department of Psychiatry and Neurochemistry, The Sahlgrenska Academy, University of Gothenburg, Mölndal, Sweden; 3 Hitachi Europe Ltd. European Rail Research Centre, Holborn, London, UK; 4 Reta Lila Research Laboratories and Department of Molecular Neuroscience, UCL, Institute of Neurology, 1 Wakefield Street, London, WC1N 1PJ, UK; 5 Department of Clinical and Experimental Epilepsy, Institute of Neurology, Queen Square, London, WC1N 3BG, UK; 6 Neurosciences Therapeutic Area, GlaxoSmithKline R&D, Gunnels Wood Road, Stevenage, SG1 2NY, UK

**Keywords:** synaptic transmission, Alzheimer’s disease, amyloid, gene expression, mouse

## Abstract

Detecting and treating Alzheimer’s disease, before cognitive deficits occur, has become the health challenge of our time. The earliest known event in Alzheimer’s disease is rising amyloid-β. Previous studies have suggested that effects on synaptic transmission may precede plaque deposition. Here we report how relative levels of different soluble amyloid-β peptides in hippocampus, preceding plaque deposition, relate to synaptic and genomic changes. Immunoprecipitation-mass spectrometry was used to measure the early rise of different amyloid-β peptides in a mouse model of increasing amyloid-β (‘TASTPM’, transgenic for familial Alzheimer’s disease genes *APP/PSEN1*). In the third postnatal week, several amyloid-β peptides were above the limit of detection, including amyloid-β_40_, amyloid-β_38_ and amyloid-β_42_ with an intensity ratio of 6:3:2, respectively. By 2 months amyloid-β levels had only increased by 50% and although the ratio of the different peptides remained constant, the first changes in synaptic currents, compared to wild-type mice could be detected with patch-clamp recordings. Between 2 and 4 months old, levels of amyloid-β_40_ rose by ∼7-fold, but amyloid-β_42_ rose by 25-fold, increasing the amyloid-β_42_:amyloid-β_40_ ratio to 1:1. Only at 4 months did plaque deposition become detectable and only in some mice; however, synaptic changes were evident in all hippocampal fields. These changes included increased glutamate release probability (*P < *0.001, *n = *7–9; consistent with the proposed physiological effect of amyloid-β) and loss of spontaneous action potential-mediated activity in the cornu ammonis 1 (CA1) and dentate gyrus regions of the hippocampus (*P < *0.001, *n = *7). Hence synaptic changes occur when the amyloid-β levels and amyloid-β_42_:amyloid-β_40_ ratio are still low compared to those necessary for plaque deposition. Genome-wide microarray analysis revealed changes in gene expression at 2–4 months including synaptic genes being strongly affected but often showing significant changes only by 4 months. We thus demonstrate that, in a mouse model of rising amyloid-β, the initial deposition of plaques does not occur until several months after the first amyloid-β becomes detectable but coincides with a rapid acceleration in the rise of amyloid-β levels and the amyloid-β_42_:amyloid-β_40_ ratio. Prior to acceleration, however, there is already a pronounced synaptic dysfunction, reflected as changes in synaptic transmission and altered gene expression, indicating that restoring synaptic function early in the disease progression may represent the earliest possible target for intervention in the onset of Alzheimer’s disease.

## Abstract (precis)

Understanding the earliest changes in Alzheimer’s disease may help in the prevention of cognitive impairment. In a transgenic mouse model, Cummings *et al.* show that synaptic changes occur shortly after soluble amyloid-β levels become measurable, and before the rapid increases in total Aβ and Aβ_42_:Aβ_40_ that lead to detectable plaque deposition.

## Graphical abstract

**Figure:**
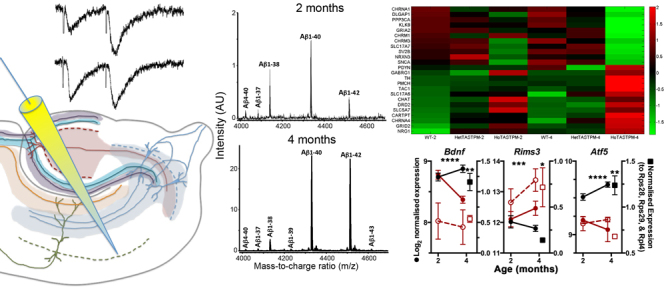
Understanding the earliest changes in Alzheimer’s disease may help in the prevention of cognitive impairment. In a transgenic mouse model, Cummings *et al.* show that synaptic changes occur shortly after soluble amyloid-β levels become measurable, and before the rapid increases in total Aβ and Aβ_42_:Aβ_40_ that lead to detectable plaque deposition.

## Introduction

Alzheimer’s disease is only diagnosed after cognitive deficits become evident late in the disease, by which stage extensive synaptic and neuronal degeneration have occurred (Fox *et al.*, 1996). Thus treatments that avoid or delay these irreversible consequences by targeting the earliest changes are urgently needed. Mice transgenic for familial Alzheimer’s disease genes do not develop tau tangles and often lack extensive neuronal loss or learning deficits. They are consequently not good models for the full course of Alzheimer’s disease (Howlett and Richardson, 2009) making the poor translation from animal models to treatments for advanced disease states unsurprising. However, transgenic mice are appropriate models for studying the effects of the initial rise of amyloid-β in the brain, which is likely one of the earliest events in all forms of the disease, regardless of the trigger initiating that rise (Jack *et al.*, 2013). Note that the initial rise of different soluble amyloid-β species and the development of plaques do not correlate with neurodegenerative changes, but rather precede this advanced stage of the disease. This period of disease progression before diagnosis lasts for decades representing a valuable window for therapeutic intervention before irreversible neurodegenerative changes and consequent cognitive loss occur (see Lesne *et al.*, 2013 for a review). In this study, we investigate the changes likely to be occurring at the earliest phases of Alzheimer’s disease in TASTPM mice as levels of amyloid-β first rise due to expression of transgenic mutant genes that clinically result in familial Alzheimer’s disease (*APP_Swe_/PSEN1_M146V_*; Howlett *et al.*, 2004). Changes preceding plaques (although without measurement of soluble amyloid-β peptides) have previously been reported in mouse models (Jacobsen *et al.*, 2006; Palop *et al.*, 2007; D'Amelio *et al.*, 2011). Recently Tozzi *et al.* (2015) also reported changes in long-term potentiation and excitatory synaptic currents preceding plaque deposition in double *APP* transgenic mice, although another study found no change in the same mice at this stage (Jolas *et al.*, 2002).

The TASTPM phenotype develops gradually, but quickly enough to make it practical for detecting early changes and for combining a multidisciplinary array of information (Howlett *et al.*, 2008). The complicating factors of single housing, generally used in past studies of *APP* transgenic mice, is avoided by using enriched environments, in which the mice can be successfully group-housed despite an aggressive phenotype. Single housing is a model for schizophrenia (Gresack *et al.*, 2010) and thus adds a potentially confounding variable, independent of amyloid-β accumulation. As environmental enrichment has been reported to rescue the phenotype of some transgenic animals (Hannan, 2014), we also confirm the histological status of TASTPM mice under the conditions of this study.

We concentrate on changes in fast synaptic transmission and differential gene expression in hippocampus of TASTPM mice, from the third week of life when we can barely detect amyloid-β, through to 4 months when plaques are first detected. As the release of amyloid-β occurs from glutamatergic neurons in an activity-dependent fashion (Kamenetz *et al.*, 2003), changes in spontaneous activity of excitatory transmission in the hippocampus might influence the amyloid-β levels (see Stargardt *et al.*, 2015 for review). We thus study the spontaneous glutamatergic activity as well as investigating whether we can detect increased release probability, this being one of the putative physiological effects of released amyloid-β (Abramov *et al.*, 2009) that could link the physiological and pathophysiological outcomes of amyloid-β release. Our recent genome-wide microarray analysis of gene expression in these mice (Matarin *et al.*, 2015) has allowed network analysis of gene expression that reveals primary changes in synaptic genes and a range of possible drug targets.

## Materials and methods

See supplementary material for further details of materials and methods used.

### Animals

All experiments were performed in agreement with the Animals (Scientific Procedures) Act 1986, with local ethical approval and in agreement with the GlaxoSmithKline statement on use of animals. Unless otherwise stated, male heterozygous TASTPM mice, bred by crossing male homozygous TASTPM (*APP_Swe_/PSEN1_M146V_*; Howlett *et al.*, 2004) with female C57Bl/6j were compared to age-matched, non-littermate male C57Bl/6j mice. Mice were group-housed in enriched environments to circumvent the aggressive TASTPM phenotype. TASTPM and wild-type mice were bred and housed in the same room under the same conditions at University College London.

### Acute hippocampal brain slice preparation

Acute brain slices were prepared using standard methods (Edwards *et al.*, 1989) adapted for mice (Parsley *et al.*, 2007). Details differing from these methods are described briefly. Following decapitation the brain was rapidly removed into ice-cold dissection artificial CSF [containing (in mM): 125 NaCl, 2.4 KCl, 26 NaHCO_3_, 1.4 NaH_2_PO_4_, 20 D-glucose, 3 MgCl_2_, 0.5 CaCl_2_, ∼315 mOsm/l, pH ∼7.4] and hippocampal slices with attached entorhinal cortex (400 µm) were cut transverse to the long axis of the hippocampus. At 5-min intervals, slices were transferred through a series of oxygenated chambers (95% O_2_/5% CO_2_) containing dissection artificial CSF (21–24°C) to artificial CSF (36°C) with consecutive Ca^2+^ and Mg^2+^ ion concentrations (in mM): (i) 1 Mg^2+^, 0.5 Ca^2+^; (ii) 1 Mg^2+^, 1 Ca^2+^; and (iii) 1Mg^2+^, 2 Ca^2+^. In the final chamber, containing recording artificial CSF (as for dissection artificial CSF but with 2 mM Ca^2+^ and 1 mM Mg^2+^), slices were allowed to return to room temperature and recover for at least 40 min before experimentation.

### Electrophysiological recordings in brain slices

Patch clamp recordings were made at room temperature (21–24°C) using CsCl-based intracellular solution. For recording field potentials, brain slices were submerged in a heated (30 ± 1°C) recording chamber, superfused with artificial CSF.

Responses were evoked by applying stimuli (100 μs) using an artificial CSF-filled glass electrode placed as indicated in Supplementary Fig. 1. Signals were filtered at 10 kHz and subsequently at 2 kHz during digitization (10 kHz). WinEDR or WinWCP (Strathclyde Electrophysiology Software) were used for detection and measurement of events. Criteria for detection of spontaneous and miniature events were to remain over a 3 pA threshold for >2 ms; rise time was <3 ms and faster than the decay. All analyses in this and all subsequent sections were carried out blind to genotype. To ensure that changes in frequency were not the result of decreased amplitude resulting in loss of events into the baseline noise, control experiments were carried out to assess the effects of decreased amplitude by decreasing the holding voltage to a level at which the mean amplitude in slices from wild-type mice was equivalent to that seen in TASPTM mice and the frequency was assessed (Supplementary Fig. 2).

### Hippocampal histology

Sections were stained using standard immunohistological techniques (Supplementary material). Primary amyloid-β_40_ or amyloid-β_42_ (1:300, Invitrogen) antibody was incubated overnight. Sections were then incubated in goat anti-rabbit secondary antibody (1:600, Jackson ImmunoResearch). For estimating neuronal loss, sections were incubated in 0.2% cresyl violet.

In each section cut across the hemisphere, the entire area of the hippocampal regions contained within that section was imaged by collaging tiled adjacent subregions. To represent data from the entire hippocampus multiple sections were imaged at different levels through the structure using an EVOS FL Auto microscope (Life Technologies). For quantification, a 480 × 360 μm region was defined in CA1, CA3 and dentate gyrus of each section. The image was converted to 8 bit, an equivalent threshold applied and each plaque traced and measured in ImageJ (NIH). For estimation of neuronal loss, neurons in each region were manually counted using ImageJ.

### Immunoprecipitation–mass spectrometry

Immunoprecipitation–mass spectrometry was performed as described in Portelius *et al.* (2010) with the following adaptions for mouse: for immunoprecipitation, 4 µg of the amyloid-β-specific antibodies 6E10 and 4G8 were separately added to 25 µl Dynabeads® M-280 (Dynal) sheep anti-mouse according to the manufacturer’s product description. Data were averaged from both hemispheres of each animal and then mean and standard error of the mean (SEM) calculated from three animals per age group.

### RNA extraction, microarrays and gene–network analysis

All procedures were completed as described in Matarin *et al.*, (2015) and genome-wide data can be found at www.mouseac.org or at http://www.ncbi.nlm.nih.gov/geo (accession GSE64398). Genome-wide microarray data were confirmed by quantitative PCR on hippocampal RNA isolated from 4-month-old homozygous TASTPM and wild-type counterparts.

### Statistical analyses

All sample sizes indicate number of animals. Where more than one sample was taken from an individual animal, data were pooled and mean SEM calculated from the individual animal means. Statistical analyses were performed using Prism (GraphPad) as indicated.

## Results

### Assessment of amyloid-β load

**Figure 1:**
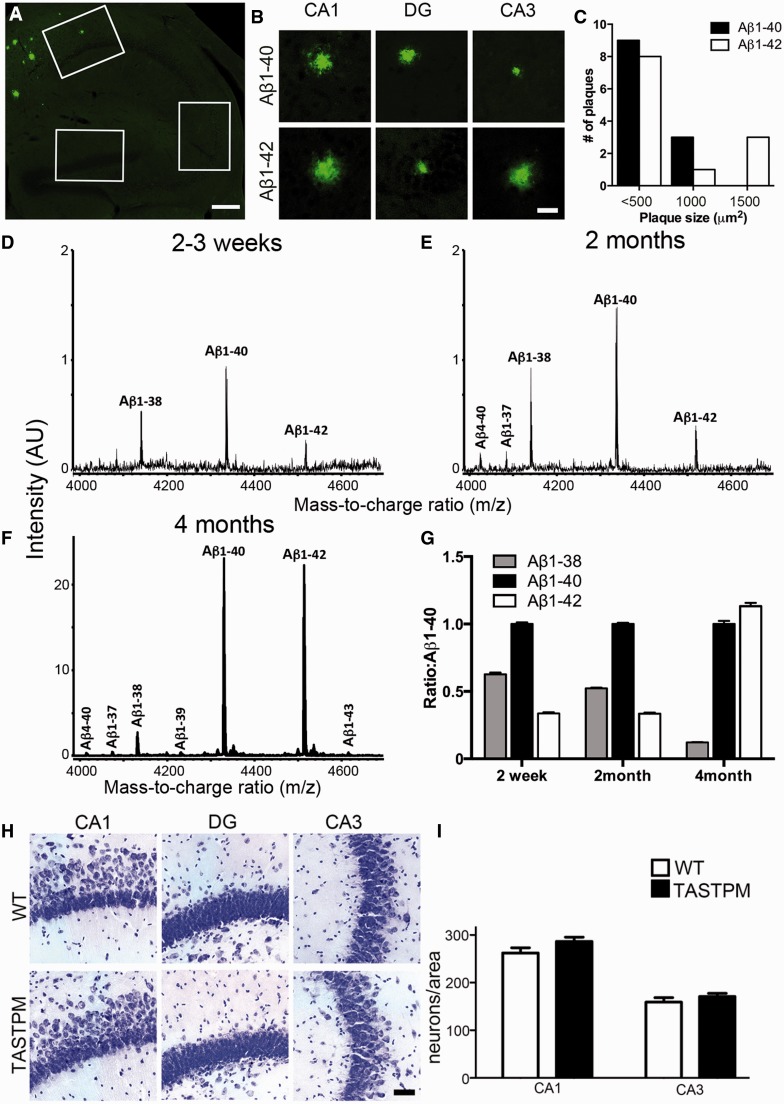
**Amyloid-β, hippocampal plaque loads and neuronal counts in 4-month-old TASTPM mice**. (**A**) Representative images of the TASTPM hippocampus immunostained with amyloid-β_40_ antibody showing spatial distribution of amyloid-β plaques. Boxes define regions used for quantification and plaque frequency distribution analysis. Scale bar = 200 μm. (**B**) Representative images of plaques observed in CA1, dentate gyrus (DG) and CA3 detected with antibodies to amyloid-β_40_ and amyloid-β_42_. Scale bar = 25 µm. (**C**) Size-frequency distribution of all amyloid-β plaques counted across all areas of hippocampi from TASTPM (plaques were detected in five of eight mice examined). All plaques were relatively small, with the majority of plaques at 4 months <500 μm^2^. (**D–F**) Representative examples of immunoprecipitation-mass spectra from TASTPM at ages indicated (*n = *3 per age and genotype). Note the different *y*-axes between **F** and **D**, (**G**) Ratios of amyloid-β isoforms (normalized to amyloid-β_40_ at each age). (**H**) Sections from 4-month-old wild-type and TASTPM mice stained with cresyl violet. Scale bar = 50 µm. (**I**) No significant neuronal loss is detected at 4 months old in either CA1 or CA3 regions of the TASTPM hippocampus, *t*-test *P* > 0.05. Data represented as mean ± SEM in a 480 × 360 µm area, *n = *3 animals wild-type; *n* = 4 animals TASTPM; the dentate gyrus was too dense for quantification. WT = wild-type.

We first assessed the plaque development under the conditions of this study, i.e. environmentally enriched group-housing (Fig. 1). At 2 months of age no amyloid-β plaques were detected in the eight TASTPM mice examined; by 4 months sparse plaques were detected in the hippocampi of five of eight TASTPM mice examined. These plaques were rare and small (<1200 µm^2^), occurring most frequently in the CA1 region of the hippocampus and the subiculum (Fig. 1A–C) but not present in every section sampled. Note that, although quantification was carried out on regions of interest at different levels through the hippocampus, within each section, the whole hippocampal region was imaged. Hence plaques outside the region of interest were also detected in the qualitative analysis of whether there were any detectable plaques.

Having established that the first plaques in TASTPM mice appear by 4 months, immunoprecipitation-mass spectrometry was used to investigate relative levels of different amyloid-β peptides at this and earlier ages before appearance of plaques.

At 2–3 weeks old, amyloid-β could already be detected in the hippocampus of TASTPM mice, with amyloid-β_38_:amyloid-β_40_:amyloid-β_42_ ratios of approximately 3:6:2 (Fig. 1D and G, *n = *3 TASTPM mice per age). Amyloid-β_4-40_ and amyloid-β_1-37_ were also detected in all samples, albeit just above the limit of detection (in the range of 250 pg/ml) and at about half the level of amyloid-β_42_. By 2 months, the peptides detected and their ratios were similar but the levels were ∼50% higher (Fig. 1E and G). Note that at this stage there were no detectable plaques and hence this presumably largely represents soluble amyloid-β.

By 4-months-old, when plaques were first detected, the situation had changed considerably. At this stage the levels increased further but the ratios of different peptides were also greatly altered. Only small increases (0–60%) in the shorter peptides were identified compared to 2 months but amyloid-β_40_ increased >7-fold and amyloid-β_42_ by ∼25-fold, reaching the same levels or even higher than amyloid-β_40_ (Fig. 1F and G). Some new peptides also reached detectable levels at this age with amyloid-β_4-40_, amyloid-β_1-39_ and amyloid-β_1-43_ also showing similar levels to the shorter peptides above, being 1–3% of the level of amyloid-β_42_. Hence the deposition of plaques coincided with a rapid change in both peptide levels and ratios, including a considerable rise in the ratio of amyloid-β_42_:amyloid-β_40_. We thus concentrated on this period of rapid change to investigate under what conditions changes in synaptic transmission became evident. No amyloid-β peptides could be detected in the wild-type mice at any age.

Despite the changes in amyloid-β levels and ratios, neuronal density remained unchanged at 4 months in both the CA1 and CA3 regions of TASTPM compared to wild-type (Fig. 1H and I).

### Synaptic transmission is changed in all hippocampal regions before amyloid-β deposits first become evident

#### CA1 region

##### Spontaneous and miniature excitatory synaptic activity

**Figure 2:**
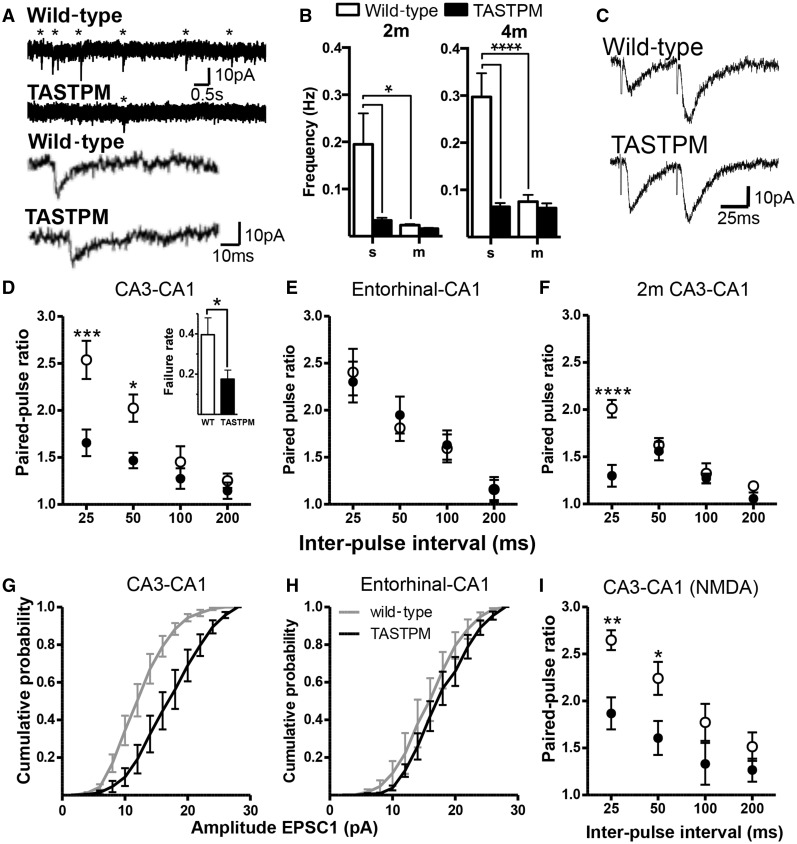
**Absence of spontaneous EPSCs in TASTPM CA1 pyramidal neurons (4-months-old unless otherwise indicated)**. (**A**) *Top*: Example traces (V_hold_ = −70 mV) showing spontaneous EPSCs from 4 m TASTPM and age-matched wild-type mice. Asterisks indicate confirmed EPSCs. *Bottom*: Example miniature EPSCs from 4 m TASTPM and wild-type mice. (**B**) Mean frequency of spontaneous (s) EPSCs and miniature (m) EPSCs. Sample sizes (WT/TASTPM): spontaneous EPSCs: 2 m: 11/7; 4 m: 7/7; miniature EPSCs: 2 m: 8/5; 4 m: 8/8 animals. Two-way ANOVA revealed a significant main effect of genotype (2 m: *P < *0.05; 4 m: *P < *0.001) and tetrodotoxin (2 m: *P < *0.05; 4 m: *P < *0.001). (**C**) Example evoked EPSCs from CA3–CA1 synapses at 4 m. (**D**) Paired-pulse ratios (EPSC2_amplitude_/EPSC1_amplitude_) were lower in TASTPM (*n = *8) than wild-type (*n = *6) mice at CA3–CA1 synapses. Two-way ANOVA: main effect of interval (*P < *0.0001); genotype (*P < *0.01). *Post hoc* significance as indicated. *Inset*: The proportion of stimuli that failed to evoke a successful EPSC was lower in TASTPM than wild-type. (**E**) Entorhinal cortical-CA1 synapses (temporoammonic pathway) showed no difference in paired-pulse ratios between wild-type (*n = *5) and TASTPM (*n = *6). (**F**) Paired-pulse ratios at CA3–CA1 synapses in 2-month-old wild-type (*n = *5) and TASTPM (*n = *5) animals. Two-way ANOVA revealed significant main effects of genotype (*P = *0.016), and interval (*P < *0.0001). (**G**) First unitary EPSCs at Schaffer collateral (CA3-CA1) synapses are larger in slices from TASTPM (*n = *6) than wild-type (*n = *8); (*P < *0.0001). (**H**) In contrast, at temporoammonic synapses, the amplitudes of the first unitary EPSCs were statistically identical between TASTPM (*n = *6) and wild-type mice (*n = *5). (**I**) Paired-pulse ratios of pharmacologically isolated NMDA receptor-mediated EPSCs at CA3–CA1 synapses were lower in TASTPM (*n = *7) than wild-type mice (*n = *6). Two-way ANOVA revealed significant main effects of genotype (*P = *0.01) and interval (*P < *0.0001). In all panels significance by Sidak *post hoc* analysis indicated as *P < 0.05, **P < 0.01, ***P < 0.001, ****P < 0.0001. WT = wild-type.

AMPA (α-amino-3-hydroxy-5-methyl-4-isoxazolepropionic acid) receptor-mediated synaptic currents were isolated using patch-clamp recordings of hippocampal CA1 pyramidal neurons in the presence of the GABA_A_ receptor antagonist gabazine (6 µM). At 4 months old, the frequency of spontaneous excitatory postsynaptic currents (EPSCs) in TASTPM was substantially lower than in wild-type mice (Fig. 2A and B; *P < *0.0001).

In a subset of slices (from three wild-type and two TASTPM mice), application of the AMPA receptor antagonist CNQX (6-cyano-7-nitroquinoxaline-2,3-dione; 20 µM) and the NMDA receptor antagonist DL-AP5 (2-amino-5-phosphonopentanoic acid; 50 µM) in the presence of gabazine abolished all spontaneous currents, confirming their glutamatergic origin (data not shown).

In other slices, to isolate miniature EPSCs, the sodium channel blocker tetrodotoxin (1 µM) was added to the gabazine solution. As expected, tetrodotoxin decreased the frequency of EPSCs by ∼3-fold in slices from 4-month-old wild-type mice (Fig. 2B). In contrast, tetrodotoxin had no significant effect on EPSCs in slices from TASTPM mice (Fig. 2B).

Considering the rapid rise in amyloid-β levels and amyloid-β_42_:amyloid-β_40_ ratios at 4 months, we went on to investigate whether this represented the first changes in synaptic transmission, suggesting a causal link. However, the change in spontaneous EPSCs was similar when the experiment was repeated at 2 months, at which stage no plaques could be found and overall amyloid-β levels and amyloid-β_42_:amyloid-β_40_ ratio was 3-fold lower (Fig. 2B). Thus, from 2 months onwards in TASTPM mice, there was an almost complete absence of spontaneous action potential-dependent EPSCs.

Consistent with this, the median amplitude of spontaneous EPSCs in 2- and 4-month-old TASTPM mice was the same as for miniature EPSCs; while in cells from wild-type mice, spontaneous EPSCs showed greater median amplitudes by ∼20% (wild-type: 13.2 ± 0.6 pA, *n = *7 animals; TASTPM 10.7 ± 0.9 pA, *n = *7 animals; *P* < 0.05).

CA1 pyramidal cells receive excitatory inputs both from CA3 neurons via Schaffer collaterals and from entorhinal cortex. However, when the Schaffer collaterals were cut in slices from wild-type mice, the frequency of spontaneous EPSCs decreased to approximately the same level as miniature EPSCs suggesting that most of the spontaneous activity originates from the CA3 cells (Supplementary Fig. 2) and hence it is this activity that is lost in TASTPM mice. Considering that we had already established that there was no loss of CA3 neurons (Fig. 1), we went on to investigate whether Schaffer collaterals remained excitable and to compare them to entorhinal inputs.

##### Minimal evoked transmission from different input pathways

In slices from 4-month-old mice, EPSCs were evoked by placing an extracellular glass electrode either in the Schaffer collaterals in stratum radiatum or amongst the entorhinal inputs in the stratum lacunosum moleculare, while making a whole-cell patch clamp recording from a CA1 pyramidal neuron. Once the minimal stimulus voltage was established, paired-pulse stimuli were applied at intervals between 25 and 200 ms. AMPA or NMDA receptor-mediated currents were isolated by including appropriate antagonists in the bath solution (as above) and altering the Mg^2+^ concentration of the artificial CSF. Note that the evoked EPSCs from wild-type mice are of similar amplitude to spontaneous EPSCs, consistent with stimulation of single or very few axons.

CA1 synapses from both Schaffer collaterals and entorhinal afferents in wild-type mice show paired-pulse facilitation (Fig. 2C), with the second EPSC in the pair having an amplitude of ∼2.5 times the amplitude of the first at the 25 ms interval, irrespective of the pathway stimulated (Fig. 2D and E). As expected, as the interval is increased, the interaction between the stimuli decreases, resulting eventually in a paired-pulse ratio close to one. At Schaffer collateral-CA1 synapses, the amplitude of the first EPSC in the pair was significantly greater in slices from TASTPM mice than from wild-type mice (Fig. 2G), demonstrated by the shift to the right in the amplitude cumulative probability plot. This could be explained by an increase in glutamate release probability, as the paired-pulse ratio was considerably lower in slices from TASTPM than from wild-type mice at the shortest intervals (Fig. 2D) and there were significantly fewer failures to release glutamate (Fig. 2D inset). These effects were specific to the Schaffer collaterals, as stimulation of the axons from entorhinal cortex showed no difference between wild-type and TASTPM mice in amplitude of evoked EPSCs in CA1 pyramidal neurons, or in paired-pulse ratios (Fig. 2E and H).

In combination these data suggest that, although the Schaffer collaterals do not fire action potentials spontaneously in TASTPM mice, they are nevertheless intact and can be activated by extracellular stimulation. Moreover, when activated, CA3 axons from TASTPM mice release neurotransmitter more readily than CA3 axons from wild-type mice, reflecting a higher probability of glutamate release. However, the change in release probability is specific to the Schaffer collaterals and does not occur in the entorhinal pathway.

Isolated NMDA receptor-mediated currents evoked by Schaffer collateral stimulation were then recorded in nominally Mg^2+^-free artificial CSF with the AMPA receptor antagonist NBQX (2,3-Dioxo-6-nitro-1,2,3,4-tetrahydrobenzo[*f*]quinoxaline-7-sulfonamide disodium salt; 10 µM) and GABA_A_ receptor antagonist, 6 µM gabazine. Consistent with the changes in AMPA receptor-mediated EPSCs described above, paired-pulse ratios of NMDA receptor-mediated EPSCs were lower in TASTPM than wild-type mice, consistent with a presynaptic change in probability of glutamate release (Fig. 2I).

Similar to the spontaneous activity, the paired-pulse ratio of AMPA receptor-mediated EPSCs was tested at 2 months and a significant decrease was already evident (Fig. 2F).

#### CA3 region

Given the substantial presynaptic changes observed at CA3-CA1 synapses, we next studied synaptic transmission in the CA3 region. In the slices used in this study, in many cases CA3 neurons tended to be too fragile for patch clamp recording in both wild-type and transgenic mice. Thus, to avoid changing the slice angle or using a selected population of cells, we used extracellular field recording for this section of the study. CA3 neurons receive inputs from different sources and the specific pathways can be evoked separately by appropriate electrode placement. Possibly due to the angle of the slice (optimized for CA1 recording) or the very proximal location of the synapses in relation to the cell soma (Brown and Johnston, 1983), especially in mouse, we were unable to evoke clear field responses when stimulating the mossy fibre pathway in our preparation. We could, however, identify CA3–CA3 synapses and perforant path–CA3 synapses, the latter originating in entorhinal cortex.

**Figure 3:**
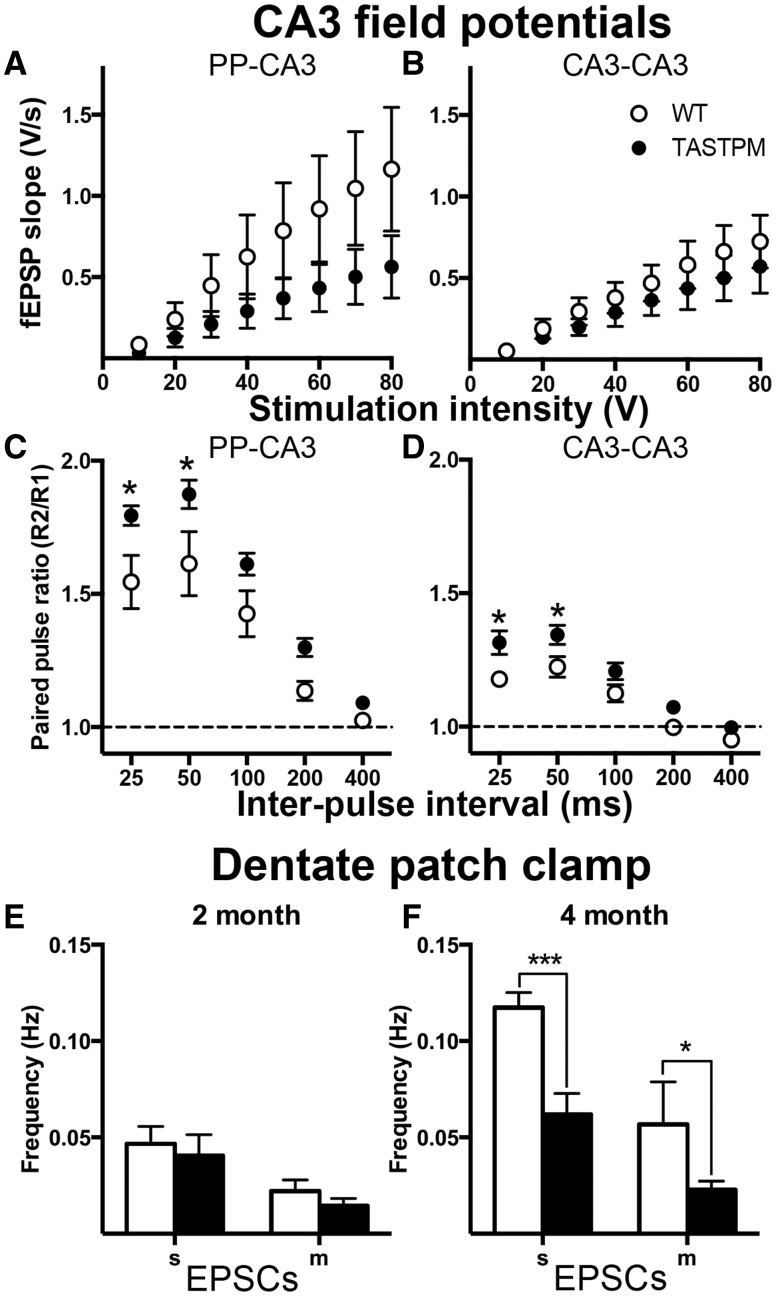
**Synaptic transmission within the TASTPM CA3 and dentate gyrus.** (**A**) Perforant path (PP)-CA3 synapses input-output relationship. (**B**) CA3-CA3 recurrent synapses input-output relationship. (**C**) Paired-pulse ratio profile at PP-CA3 synapses. (**D**) Paired-pulse ratio profile at CA3–CA3 synapses. Sample sizes (wild-type/TASTPM) **A** and **C**: 7/8; **B** and **D**: 7/7. (**E**) Spontaneous EPSC frequencies in 2 month dentate gyrus. Sample sizes (wild-type/TASTPM): spontaneous EPSCs: 10/7; miniature EPSCs 7/8 animals. (**F**) Miniature EPSC frequencies in 2 month dentate gyrus. Sample sizes (wild-type/TASTPM): spontaneous EPSCs: 9/9; miniature EPSCs 6/8 animals. Two-way ANOVA: main effects of genotype (*P < *0.0001) and tetrodotoxin (*P < *0.0001).

In both pathways, although there was no significant difference in input/output relationships between genotype, the TASTPM responses tended to be smaller than wild-type (Fig. 3A and B). Moreover, in contrast to the CA1 region, TASTPM mice displayed greater paired-pulse ratios than wild-type (Fig. 3C and D). Two-way ANOVA revealed a significant main effect of both genotype (*P < *0.05) and interval (*P < *0.0001); *post hoc* multiple comparisons revealed significant differences between wild-type and TASTPM at both 25 and 50 ms interpulse intervals.

The increase in paired-pulse ratio suggests a decreased probability of glutamate release in both pathways and, although the trend to a difference in input/output relationship did not reach statistical significance, this would be consistent with a decrease in activity in the CA3 region, which would likely result in a decrease in spontaneous activity in the CA1 region, as reported above. Note, however, that the interpretation of this result should be treated with some caution, as the conditions of a field recording in the absence of antagonists is somewhat different from the situation of recording minimal responses from a single neuron in the presence of a GABA_A_ antagonist and may have been influenced by unassessed factors in the synaptic network.

#### Dentate gyrus

**Figure 4:**
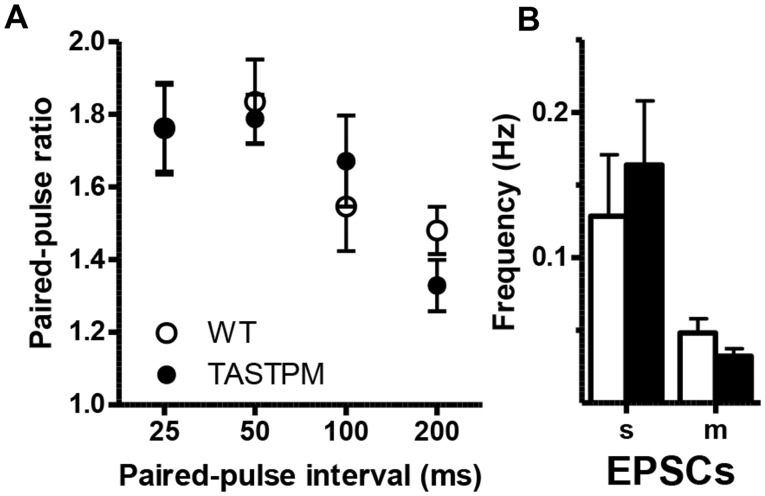
**Normal synaptic transmission at TASTPM CA3-CA1 synapses at 2 weeks of age.** (**A**) Paired-pulse ratios at CA3-CA1 synapses from 2-week-old TASTPM are normal. (**B**) No difference between TASTPM and wild-type spontaneous (s) or miniature (m) EPSCs recorded from CA1 pyramidal neurons from 2-week-old TASTPM. (Wild-type: *n = *5 animals; TASTPM, *n = *5 animals.).

Mossy fibres arise from dentate gyrus granule cells and so we made patch clamp recordings of spontaneous synaptic currents from these cells. While no changes were observed in the frequency of spontaneous EPSCs or miniature EPSCs at 2 months (Fig. 3E), the frequency of spontaneous EPSCs was lower in cells from 4-month-old TASTPM mice than wild-type (Fig. 3F). However, unlike in the CA1 region, in the presence of tetrodotoxin (1 µM), the frequency of miniature EPSCs was lower in TASTPM than wild-type mice (Fig. 4F) and miniature EPSC amplitude also tended to be lower, although this did not reach significance (8.9 ± 0.7 pA, *n = *7 animals and 7.4 ± 0.3 pA, *n = *8 animals, respectively; *P = *0.06). This suggests a change in postsynaptic receptors as well as release probability or number of release sites of the perforant path axons.

In summary, at 4 months old, excitatory transmission was affected in all parts of the hippocampal tri-synaptic circuit, with the frequency of spontaneous EPSCs being decreased throughout, as measured in the CA1 and dentate gyrus and as inferred from release probability or activity of the incoming pathways in the CA3 region. No neuronal loss was evident. Release probability of inputs onto CA1 pyramidal cells from the CA3 terminals was increased, as was release probability from the perforant path onto dentate granule cells. However, in contrast, the release probability of at least two of the pathways impinging on CA3 cells seemed to be decreased, suggesting that this could be a major factor in the decreased spontaneous activity seen in the CA1 region. Although the changes measured in the dentate gyrus were only seen at 4 months, the changes in the CA1 region did not depend on the high levels of amyloid-β detected at 4 months, nor on a high ratio of amyloid-β_42_:amyloid-β_40_ as similar changes were seen already at 2 months of age.

### Changes in synaptic transmission precede sharp rises in amyloid-β and plaque deposition but are not developmental

Considering the large synaptic changes seen in 2-month-old TASTPM mice in the CA1 region, despite the lack of plaque deposition, one possibility was that the effects seen were developmental, due to the position of transgene insertion or overexpression of the *APP* and/or *PSEN1*, independent of increasing amyloid-β levels. We thus tested whether we could detect synaptic changes in the CA1 region in mice at 2-3 weeks old. No changes were detectable in spontaneous, miniature or evoked EPSCs (Fig. 4). We also measured synaptic transmission in slices from TAS10 mice that express the APP transgene alone and found no significant changes up to 4 months (Supplementary Fig. 3). Hence we conclude that the changes detected at 2 months in TASTPM mice are some of the earliest changes occurring and result from the slowly increasing levels of soluble amyloid-β that are insufficient, or have not been present for long enough, to have measureable effects at 2–3 weeks.

### Genome-wide expression analysis in 2- and 4-month-old mice shows strong changes in synaptic genes

To investigate gene expression changes that might be associated with the early synaptic changes, we accessed and extended the relevant sections of a genome-wide microarray study that we have recently undertaken on this and other transgenic mouse models that express human genes for dementia (Matarin *et al.*, 2015). Weighted Gene Co-expression Network Analysis (WGCNA) was applied to genes with highly variable expression at 2 and 4 months to identify novel modules of genes with co-expression and summarize the main enriched biological functions and molecular pathways that are disrupted at these early stages in TASTPM mice. To increase the variability of gene expression and effectively bring in a dose-dependence to the analysis, we compared the wild-type gene expression to both heterozygous and homozygous TASTPM mice.

**Figure 5:**
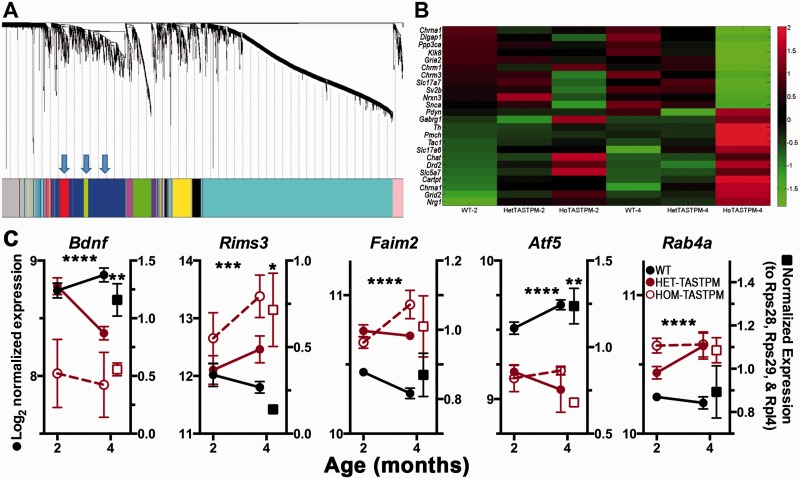
**Analysis of gene co-expression in hippocampus and the relation of specific synaptic modules of genes to genotype.** (**A**) Hierarchical clustering dendrogram for the most varying genes in homozygous TASTPM, heterozygous TASTPM mice and wild-type controls at 2 and 4 months old and equivalent module assignment colours. The modules that correlate with genotype are indicated with arrows. The *y*-axis corresponds to the distance determined by the extent of topological overlap. (**B**) Heat map of genes involved in synaptic transmission (GO category: GO0007268) identified by the DAVID database from the blue module of genes indicating how expression of these genes is increased or decreased in homozygous and heterozygous TASTPM mice at 2 and 4 months old in the hippocampus compared to wild-type mice. Colours represent the *z*-score of the expression level for each gene (red is high expression and green is low expression). (**C**) Further examples of expression in early differentially expressed genes at 2 and 4 months old in heterozygous (HET) or homozygous (HOM) TASTPM mice. In all cases two-way ANOVA indicated a main effect of genotype as indicated by stars. On the right of each graph the symbols represent the results of quantitative PCR analysis in the same 4-month-old animals. The same trends are seen in all cases with significant differences indicated with asterisks above the symbols. *P < 0.05; **P < 0.01; ***P < 0.001; ****P < 0.0001. Sample sizes: microarrays (WT/HET-TASTPM/HOM-TASTPM), 2 months: *n = *11/4/4; 4 months: *n = *12/4/4 mice. qPCR: *n = *4 HOM-TASTPM mice and *n* = 4 wild-type mice. WT = wild-type.

The most variable genes (∼3000 genes) were determined by their coefficient of variation, without reference to phenotype or gene function. A dendrogram of the modules of gene co-expression following WGCNA gave three modules of genes that correlated significantly with genotype: blue, red and green-yellow in Fig. 5A. The red and blue modules showed similarly high correlations with genotype (0.54, *P < *0.0005; 0.49, *P < *0.001, respectively) but while synaptic genes were included in both, they were more highly enriched in the blue module. The four most significant Gene Ontology (GO) categories in this module (Table 1) were designated ‘synaptic transmission’; ‘transmission of nerve impulses’; ‘cell-cell signalling’ and ‘cell communication’ (each with FDR < 6 × 10^−6^) which fits closely with the main changes seen in the synaptic data above, particularly changes in release probability and loss of spontaneous action potentials. This module contained various genes already associated with Alzheimer’s disease, as well as a number of novel highly differentially expressed genes. The differential expression of the genes belonging to the blue module falling into the most significant Gene Ontology category are plotted in the heat map in Fig. 5B.

**Table 1:** Top four GO categories for the ‘Synaptic' Blue module

Term	Count	%	Genes	Pop hits	Fold-change	Bonferroni	Benjamini	FDR
GO:0007268 ‘synaptic transmission’	24	6.05	*Gabrg1*, *Klk8*, *Dlgap1*, *Drd2*, *Pmch*, *Nrxn3*, *Th*, *Snca*, *Tac1*, *Pdyn*, *Slc17a7*, *Slc17a6*, *Gria2*, *Chrm3*, *Chrm1*, *Grid2*, *Chrna4*, *Cartpt*, *Sv2b*, *Slc5a7*, *Ppp3ca*, *Nrg1*, *Chrna1*, *Chat*	168	5.61	7.54 × 10^−8^	7.54 × 10^−8^	6.71 × 10^−8^
GO:0019226 ‘transmission of nerve impulse’	25	6.30	*Drd2*, *Snca*, *Th*, *Tac1*, *Grid2*, *Chrna4*, *Sv2b*, *Ppp3ca*, *Pou3f1*, *Nrg1*, *Chrna1*, *Chat*, *Gabrg1*, *Dlgap1*, *Nrxn3*, *Pmch*, *Pdyn*, *Gria2*, *Slc17a7*, *Slc17a6*, *Chrm3*, *Chrm1*, *Klk8*, *Cartpt*, *Slc5a7*	212	4.63	1.55 × 10^−6^	7.76 × 10^−7^	1.38 × 10^−6^
GO:0007267 ‘cell-cell signalling'	28	7.05	*Drd2*, *Snca*, *Th*, *Tac1*, *Shh*, *Bdnf*, *Grid2*, *Chrna4*, *Sv2b*, *Rapgef4*, *Ppp3ca*, *Nrg1*, *Chrna1*, *Chat*, *Gabrg1*, *Dlgap1*, *Klk8*, *Nrxn3*, *Pmch*, *Dlgap2*, *Pdyn*, *Slc17a7*, *Slc17a6*, *Gria2*, *Chrm3*, *Chrm1*, *Cartpt*, *Slc5a7*	276	3.99	3.52 × 10^−6^	1.17 × 10^−6^	3.13 × 10^−6^
GO:0007154 ‘cell communication'	35	8.82	*Drd2*, *Th*, *Snca*, *Itgb4*, *Tac1*, *Shh*, *Bdnf*, *Grid2*, *Chrna4*, *Sv2b*, *Rapgef4*, *Ppp3ca*, *Pou3f1*, *Chrna1*, *Nrg1*, *Chat*, *Gabrg1*, *Pdyn*, *Dlgap1*, *Atg9b*, *Klk8*, *Slc8a2*, *Nrxn3*, *Pmch*, *Dlgap2*, *Nr4a2*, *Slc17a7*, *Slc17a6*, *Gria2*, *Chrm3*, *Sfrp2*, *Chrm1*, *Avpr1a*, *Cartpt*, *Slc5a7*	428	3.21	6.38 × 10^−6^	1.60 × 10^−6^	5.68 × 10^−6^

FDR = false discovery rate.

Expression of other example genes that are strongly differentially expressed from early ages are shown in Fig. 5C for hippocampus. In each case the microarray data were validated using quantitative PCR and similar trends were apparent. It should be noted that, in each case, expression of either *APP_Swe_* (‘TAS10’) or *PSEN1_M146V_* (‘TPM’) alone, had little effect on gene expression compared to wild-type at 2 or 4 months (see www.mouseac.org; Matarin *et al.*, 2015), indicating that the changes observed are not due to the expression of the individual transgenes. Of particular interest we highlight genes, such as *Bdnf*, that have frequently been associated with Alzheimer’s disease (Lu *et al.*, 2014) and other novel genes that warrant further study as targets for early disease intervention or detection. Some of these genes code for synaptic proteins such as the active zone protein *Rims3* (Wang and Sudhof 2003), which has been associated with autism (Kumar *et al.*, 2010) and schizophrenia (Weidenhofer *et al.*, 2009). Interestingly, both *Rims3* and *Bdnf* are only increased in expression in heterozygous TASTPM mice by 4 months (although earlier in homozygous TASTPM mice) and so may be a result of the initial synaptic changes associated with rising amyloid-β rather than a cause. In contrast, *Faim2*, which encodes an anti-apoptotic protein (Somia *et al.*, 1999) shows increased expression already at 2 months, whereas *Atf5*, which has been also suggested to be neuroprotective and to regulate stem cell differentiation (Torres-Peraza *et al.*, 2013) is decreased in expression by this time. Another area of interest is the early increase of endosomal pathway gene expression, such as *Rab4,* involved in recycling receptors from the membrane to the endosome (Daro *et al.*, 1996)*.* Other small GTPases have previously been linked to Alzheimer’s disease, including an involvement of *Rab6* in amyloid precursor protein processing (McConlogue *et al.*, 1996), but *Rab4a* has not been previously implicated.

## Discussion

Here we have presented a multidisciplinary study of the earliest changes that occur in the TASTPM mouse model of rising amyloid-β in the brain, allowing us to relate rises of different amyloid-β peptides to the first synaptic and genetic changes in the hippocampus. We find a pronounced synaptic phenotype that begins as early as 2 months, when amyloid-β levels are rising in the hippocampus but clearly prior to the deposition of amyloid-β into plaques. Moreover these changes precede the later dramatic rise in amyloid-β_42_, generally suggested to be the most toxic form (Walsh and Selkoe, 2007). The fact that plaque deposition coincides with this rise in both amyloid-β levels and the ratio of amyloid-β_42_ to other amyloid-β peptides, is consistent with a recent study demonstrating that while amyloid-β_40_ or amyloid-β_42_ can alone start to cause depositions in neurites in cultures, the rate of deposition accelerates when the two peptides are presented at a 1:1 ratio (Johnson *et al.*, 2013). However, the start of synaptic changes is clearly not dependent on this. The changes feature an almost complete absence of spontaneous action potential-driven glutamate release from presynaptic glutamatergic terminals and a concurrent increase in the probability of successful glutamate release when action potentials are evoked in the Schaffer collaterals or in the perforant path to dentate gyrus. This change in release probability is, however, not uniform across all axons of the hippocampus, as entorhinal cortical axons to CA1 neurons are unaffected whereas field recordings from CA3–CA3 axons and the perforant path to CA3 axons suggest the opposite effect, with decreased release probability.

The lack of detectable change at pre-weaning stages when ratios of amyloid-β peptides are similar to those at 2 months and the overall levels are only slightly lower suggests that it is time of exposure, rather than concentration or ratio that is required. The question arises as to which of these many changes is primary and which are reactions to the initial change, either homeostatic or pathological. Physiologically, amyloid-β is proposed to be released during synaptic transmission by a process involving exocytosis, either in an activity-dependent or activity-independent manner (Cirrito *et al.*, 2008; Lundgren *et al.*, 2014). Once released, amyloid-β has been reported to increase neurotransmitter release probability in normal rodents (Abramov *et al.*, 2009). Hence it seems likely that, as amyloid-β levels begin to rise in TASTPM mice, the first effects might be increased release probability in the areas where amyloid-β is highest. This suggests the hypothesis that the decreases in spontaneous activity, seen both in terms of spontaneously occurring action potentials and release in the CA3 region, might be a series of homeostatic reactions to the initial increases in release probability in the CA1 region. The specificity to particular pathways may relate to localization of release of amyloid-β from particular cell types or to susceptibility of particular types of neurons to its effects. Exactly what effect these various changes (increased probability of transmitter release and decreased spontaneous activity in different regions) would have on the overall activity of the hippocampus *in vivo* cannot be assessed from a hippocampal slice. However, neuronal hyperactivity has been reported in early Alzheimer’s disease (see Stargardt *et al.*, 2015 for a review).

It is interesting to note that, while multiple forms of amyloid-β can be detected at these early stages in the hippocampus of TASTPM mice, it is only well after synaptic changes have begun that the amyloid-β_42_:amyloid-β_40_ ratio reaches close to or indeed over 1:1. Recently Dolev *et al.* (2013) reported that neuronal firing frequencies and patterns can alter the relative levels of different forms of amyloid-β. They report that increased firing frequencies tended to increase amyloid-β_40_ relative to amyloid-β_42_. The relative effects of loss of spontaneous activity versus increased release probability could have different effects depending on the environmental stimulus in different pathways and from moment to moment. Absence of spontaneous action potentials, such as we report here, could result in an overall lower frequency of firing in some axons and, in turn, a higher amyloid-β_42_:amyloid-β_40_ ratio. Thus the increase in amyloid-β_42_ relative to amyloid-β_40_ could also be a result of the chain of synaptic changes, eventually leading to plaque deposition.

The present study confirms that early changes in synaptic transmission precede plaque deposition as reported previously (Jacobsen *et al.*, 2006; D'Amelio *et al.*, 2011; Tozzi *et al.*, 2015) although the exact nature of such changes shows some differences between studies, probably reflecting differences in recording conditions. The confirmation of such early synaptic change in different mouse models of raised amyloid-β greatly strengthens the significance of these studies and is in agreement with the suggestion that in Alzheimer’s disease, synaptic derailment is likely to occur long before symptom onset and these studies would suggest, also prior to plaque deposition.

The development of treatments targeting these early synaptic changes could help to prevent a chain of events leading to on-going damage. To this end we have studied the earliest gene expression changes that could be associated with the synaptic phenotype. Although synaptic genes are the most consistently affected functional group, in TASTPM mice, these alterations in gene expression frequently become detectable subsequent to the changes in synaptic transmission and thus effects on synaptic gene expression may be the result, rather than the cause, of the synaptic changes. Presumably these reactive changes contribute to the on-going efficacy of cognitive function, despite the substantial changes in the synaptic network but may also contribute to the other behavioural changes seen, such as an increase in aggression and anxiety and lack of motivation (data not shown, but see Pugh *et al.*, 2007), which could relate to early symptoms reported in Alzheimer’s disease (Robins Wahlin and Byrne 2011; Ramakers *et al.*, 2013). In contrast, there are also several genes that are already considerably increased in expression at 2 months old, even in the heterozygous TASTPM mice in which the synaptic changes have been measured. Note that these genes may be either contributing to, or protecting against, the on-going changes we record and the possibility of protective rather than harmful effects must be a consideration in developing studies to test how such genes and their products could be targeted to alter the early effects of rising amyloid-β. In either case they suggest both novel drug targets and also possible novel candidates for CSF markers for the earliest stages of Alzheimer’s disease.

In summary, Alzheimer’s disease is a devastating condition. Understanding and detecting the earliest changes that occur as amyloid-β begins to rise is the only possibility for developing methods for controlling these changes and preventing the on-going chain of events that lead to neurodegeneration and cognitive impairment. The present study confirms that changes in synaptic transmission precede plaque deposition but also that they precede and may even cause the increase in the ratio of amyloid-β_42_:amyloid-β_40_, which is possibly responsible for triggering this deposition and for further toxic change. Moreover, the early changes in gene expression that accompany these synaptic changes suggest a range of possible areas for targeting drug development. The balance between regulators of apoptosis may be key at the very start in avoiding the advent of cell death or maintaining synapse number (Sheng *et al.*, 2012 for review). In contrast, synaptic gene expression is affected early but probably as a result of the initial changes in synaptic transmission, which may be caused directly by increasing amyloid-β levels (Abramov *et al.*, 2009).

## Funding

D.M.C., M.Y. and R.S. funded by Medical Research Council, UK Grant to F.A.E. (Ref: MR/J011851/1); D.A.S. funded by Alzheimer’s Research UK Senior Fellowship; GlaxoSmithKline funded the initial stages of the project. J.H. gratefully acknowledges the International Foundation for Research on Alzheimer's Disease.

## Conflict of interest

Initial studies were funded by a grant from GlaxoSmithKline to F.A.E. and continue under an MTA agreement with GlaxoSmithKline for use of mice. J.C.R. is employed by GlaxoSmithKline.

## Supplementary material

Supplementary material is available at *Brain* online.

## Abbreviations

AMPA: α-amino-3-hydroxy-5-methyl-4-isoxazolepropionic acid
CA: cornu ammonis
EPSC: excitatory postsynaptic current
NMDA: *N*-methyl D-aspartate
